# Intensity of Left Atrial Spontaneous Echo Contrast as a Correlate for Stroke Risk Stratification in Patients with Nonvalvular Atrial Fibrillation

**DOI:** 10.1038/srep27650

**Published:** 2016-06-09

**Authors:** Yuanping Zhao, Lijing Ji, Jian Liu, Juefei Wu, Yan Wang, Shuxin Shen, Shengcun Guo, Rong Jian, Gangbin Chen, Xuan Wei, Wangjun Liao, Shelby Kutty, Yulin Liao, Jianping Bin

**Affiliations:** 1 State Key Laboratory of Organ Failure Research, Department of Cardiology, Nanfang Hospital, Southern Medical University, Guangzhou, 510515, China; 2 Department of Gerontology, Cangzhou Central Hospital, Cangzhou, china; 3 Department of Ultrasonography, Nanfang Hospital, Southern Medical University, Guangzhou, 510515, China; 4 Department of Oncology, Nanfang Hospital, Southern Medical University, Guangzhou, 510515, China; 5 University of Nebraska Medical Center, Children's Hospital and Medical Center, Omaha, NE, United States

## Abstract

The intensity of left atrial spontaneous echo contrast (LASEC) by transesophageal echocardiography (TEE) has been proposed as an important variable in the stratification of thromboembolic risk, particularly in patients with nonvalvular atrial fibrillation (NVAF). We hypothesized that the quantification of LASEC by ultrasound will improve its utility in predicting subsequent stroke events in patients with NVAF. Patients (n = 206) with definite NVAF receiving TEE were included for this prospective cohort study. Baseline clinical risk factors of stroke, CHADS_2_ score and CHA_2_DS_2_-Vasc, left atrial thrombus (LAT), the five-grades of LASEC and video intensity (VI) value of LASEC were measured. During 2 years follow-up, 20 patients (9.7%) developed stroke. VI value of LASEC in the patients with stroke was higher compared to patients without stroke (25.30 ± 3.61 *vs.* 8.65 ± 0.81, p < 0.001). On logistic regression analysis, LAT, qualitative LASEC, graded LASEC, VI value of LASEC and CHADS_2_ and CHA_2_DS_2_-Vasc score were independent predictors of stroke. Among them, the highest area under the curve of receiver operating characteristic (ROC) in predicting stroke was VI value of LASEC (p < 0.05). These results show that quantification of LASEC by VI value is the most favorable predictor of stroke in patients with NVAF, and calls for improving the utility of LASEC in predicting subsequent stroke events.

Nonvalvular atrial fibrillation (NVAF) is a major risk factor for stroke and systemic embolism^1^,^2^. The estimated prevalence of atrial fibrillation (AF) in the United States is 2.7 million to 6.1 million^3^,^4^. Risk stratification schemes such as the CHADS_2_ (congestive heart failure, hypertension, age ≥75 years, diabetes mellitus, stroke) and CHA_2_DS_2_-Vasc (congestive heart failure, hypertension, age ≥75 years, diabetes mellitus, stroke, vascular disease, age 65–74 years, sex category) have been proposed for identification of NVAF patients at higher thromboembolic risk and are more likely to achieve net clinical benefit from anticoagulation^5^. However, these score has the limitation of classifying excessive number NVAF patients as at intermediate risk^6^.

Thrombogenic milieu in the left atrium (LA) is an important factor in the pathogenesis of NVAF-related thromboembolic events^7^. Presence of thrombus, smoke, or sludge in the LA, and decreased LA appendage emptying velocity has been reported as predictors of systemic embolic events^8^,^9^,^10^. Left atrial spontaneous echo contrast (LASEC) has been a common phenomenon that is detected by transesophageal echocardiography (TEE) in patients with NVAF^11^. Its formation is attributed to abnormal blood stasis, abnormal hemostasis, platelets, and fibrinolysis^12^ which are all recognized by Virchow’s triad as the pathogenesis of thrombus formation in AF^13^. Thus, LASEC has received increasing attention as a marker of thromboembolic risk in NVAF^9^. Not merely the presence or absence, but also the severity of LASEC by semi-quantitative assessment has been proven to be associated with stroke events in patients with NVAF^14^,^15^. While a few studies have attempted to correlate semi-quantitative LASEC (five-grade method) with quantitative video intensity (VI) measurements, the semi-quantitative assessment is largely subjective, influenced by the experience of the operator. Furthermore, there have been no studies to date examining the utility of a quantitative method to evaluate the severity of LASEC in predicting stroke events. Therefore, in this study, we quantified the severity of LASEC with VI value using color-coded technology. Our goal was to prospectively determine the VI value of LASEC as a quantitative stratification method, on the subsequent occurrence of stroke events in patients with NVAF.

## Results

### Patient population and baseline characteristics

**Figure 1:**
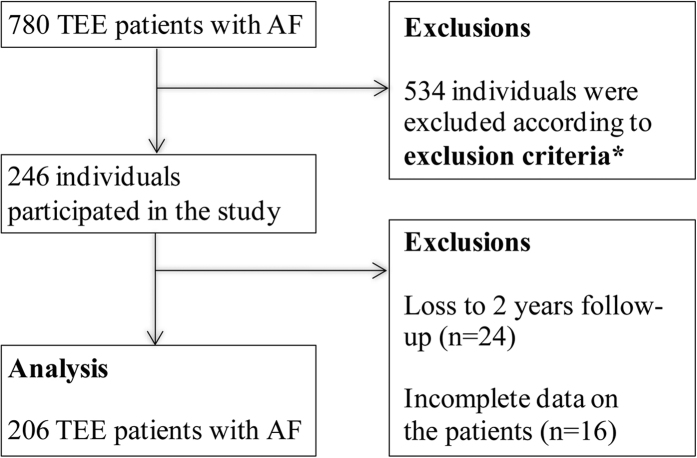
Diagram of inclusion and exclusion of the study population. TEE, transesophageal echocardiography. AF, atrial fibrillation. *Exclusion criteria (see text for details).

Among the 780 recruited patients, 246 were enrolled in the cohort study based on exclusion criteria. Of 246, 24 patients were lost to follow-up and 16 patients had incomplete data, leaving 206 patients available for analysis (Fig. 1). Table 1 presents the demographic data and clinical characteristics of the patients. Patients in each group were similar with respect to age, gender, and prevalence of hypertension, diabetes, hyperlipidemia and smoking. Twenty patients developed stroke during the 2 years follow-up. Patients who developed stroke had a higher detection rate of LASEC (75% vs. 36.6%, p = 0.001) and left atrial thrombus (LAT) (35% vs. 5.4%, p < 0.001), and higher CHADS_2_ score (1.5 ± 1.1 vs. 0.87 ± 0.85; p = 0.022, 95% CI 0.10 to 1.16) and CHA_2_DS_2_-Vasc (2.65 ± 1.42 *vs.*1.58 ± 1.25; p < 0.001, 95% CI 0.49 to 1.66).

Among the 206 patients, left atrial thrombus (LAT) was observed in 7 patients with stroke and in 10 without stroke, all of those patients received anticoagulation after the TEE study, if they had not before. Anticoagulation therapy was unchanged in patients without a LAT.

### Quantification of LASEC with color-coding and VI value

**Figure 2:**
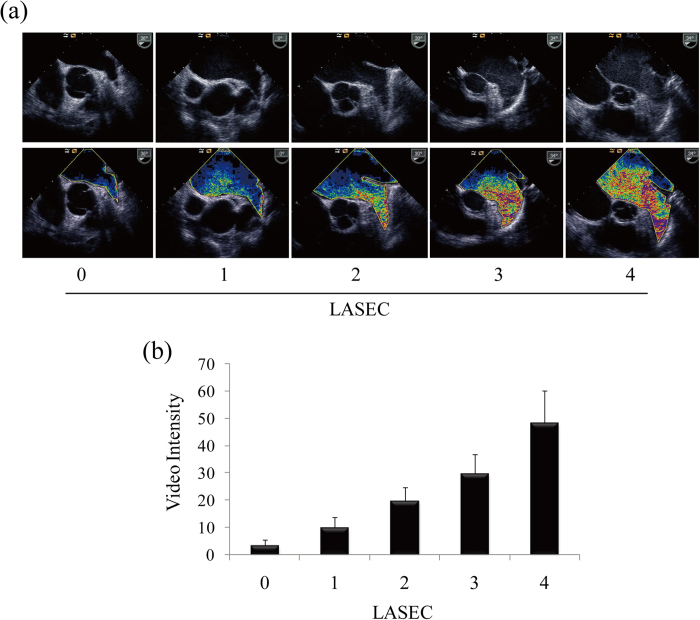
Two-dimensional ultrasonogram and the corresponding color coded map of different grades of LASEC (**a**) Quantification of VI value in patients with each of five grades of LASEC (**b**) *p < 0.05.

Examples of 5 grades of LASEC with the use of color-coded method or not are shown in Fig. 2a. Obviously, by using the color-coded method, differences between five grades of LASEC was more visible. There were 123 patients with grade 0, 38 patients with grade 1, 21 patients with grade 2, 13 patients with grade 3, and 11 patients with grade 4, respectively.

Figure 2b shows the VI value of each grade of LASEC. A strong correlation was observed between the quantitative parameter (VI value) and the semi-quantitative grading of LASEC (r^2^ = 0.857, p < 0.001). From grade 0 to grade 4, the mean VI value was 3.33 ± 2.07 (95% CI 2.96 to 3.96), 9.84 ± 3.98 (95% CI 8.54 to 11.15), 19.71 ± 5.14 (95% CI 17.37 to 22.05), 29.77 ± 7.08 (95% CI 25.05 to 34.04), and 48.27 ± 11.87 (95% CI 40.3 to 56.24), respectively. One-way ANOVA with multiple comparisons showed that there were significant differences in VI value among each grade of LASEC (p < 0.05).

### Relation between stroke incidence and grades of LASEC, or its VI value

**Figure 3:**
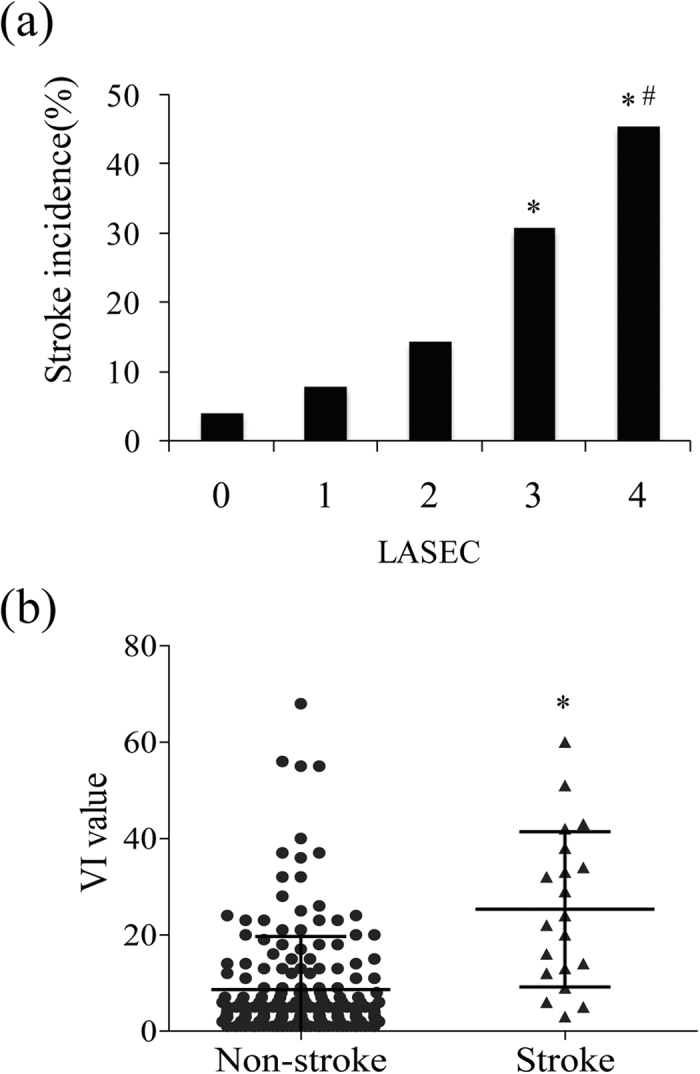
Relation between stroke incidence and grades of LASEC, or its VI value. (**a**) Stroke incidence of patients with different grades of LASEC; *p < 0.05 *vs.* grade 0, ^#^p < 0.05 *vs.* grade 1. (**b**) VI value distribution of patients with or without stroke *p < 0.01.

To understand how LASEC influences stroke risk, we first examined the stroke incidence rates for five grades of LASEC. In participants without LASEC (grade 0), stroke incidence was low (4.1%; Fig. 3a). Stroke incidence significantly increased with the severity of LASEC (7.9% for grade1, 14.3% for grade 2, and 30.8% for grade 3, respectively), reaching an incidence rate of 45.5% for grade 4 (Fig. 3a). The distribution of VI value in the stroke and non-stroke groups is illustrated in Fig. 3b. The stroke group was found to have a tendency toward higher VI values (25.30 ± 3.61 *vs.* 8.65 ± 0.81, p < 0.001, 95% CI 8.96 to 24.33).

### Independent predictors of stroke

Multivariate logistic regression analysis was performed to determine the relative importance of independent predictors of stroke (Table 2). Multiple candidate clinical predictors and echocardiography measurements were assessed as univariate independent predictors for stroke. Qualitative LASEC (p = 0.014, OR 6.59, 95% CI 1.46 to 29.71), LAT (p = 0.007, OR 6.28, 95% CI 1.66 to 23.79) and VI value of LASEC (p = 0.002, OR 1.09, 95% CI 1.03 to 1.15) were independent predictors of stroke. For graded LASEC, grade 3 (p = 0.004, OR 45.32, 95% CI 3.37 to 608.69) and grade 4 (p = 0.002, OR 157.98, 95% CI 6.05 to 4127.14) also were independently predictive of stroke, but grade 0 to 2 were not. For CHADS_2_, model 1 did not show that the CHADS_2_ was an independent predictor of stroke (p = 0.09). Both the CHADS_2_ and a subgroup of a high CHADS_2_ score of 3 were noted as an independently predictive of stroke (p = 0.044), when LASEC was taken into consideration. If considering CHA_2_DS_2_-Vasc instead of CHADS_2_, the results of multivariate logistic regression analysis was similar to CHADS_2_ (Supplementary Table S1).

### Receiver operating characteristic (ROC) curves

**Figure 4:**
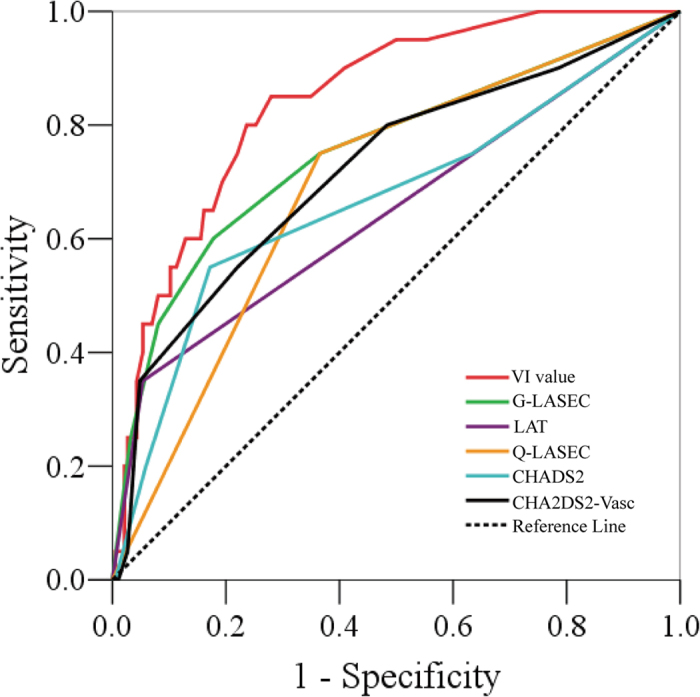
ROC curves analysis of VI value, Graded-LASEC (G-LASEC), CHA_2_DS_2_-Vasc, qualitative LASEC (Q-LASEC), LAT and CHADS_2_ in predicting the risk of stroke in patients with NVAF.

Figure 4 displays the ROC curves of VI value of LASEC, graded LASEC, CHA_2_DS_2_-Vasc, qualitative LASEC, LAT and CHADS_2_ in predicting stroke in patients with NVAF. It shows the superiority of the VI value at predicting stroke compared to the other data (p < 0.05). The sensitivity of VI value in predicting stroke was 80%, while the specificity was 76.3% with a cutoff value of 11.5. The area under the curve was 0.844 with a standard error of 0.041 (Table 3). For graded LASEC, CHA_2_DS_2_-Vasc, qualitative LASEC, CHADS_2,_ and LAT, the area under the curve was 0.754, 0.720, 0.692, 0.668 and 0.648, respectively.

## Discussion

The present study is the first of its kind to demonstrate that quantification of LASEC by VI value can predict stroke events and that it is an independent predictor of stroke in NVAF patients. Previous studies have evaluated the predictive value of LASEC based on its presence or not. In a prospective study of patients with NVAF, Sydney *et al.* observed an annual stroke event rate of 9% in patients with LASEC, as compared to only 3% in patients without LASEC^9^. Similar to our results, the stroke patients had a significant higher incidence of LASEC than the non-stroke patients. However, the intensity of LASEC may vary continuously, from sparse and barely perceptible to dense, and the magnitude of thromboembolic risk may also vary with the severity of LASEC^15^,^16^. Thus it became apparent that there is a strong correlation between stroke event rate and the severity of LASEC^17^,^18^. This was further corroborated by the development of semi-quantitative methods, including the three-grade method as Beppu *et al.* proposed and the five-grade method as Fatkin *et al.* proposed^16^,^19^. Their results revealed that denser LASEC was accompanied by a higher risk of left atrial appendage thrombus formation in patients with NVAF. In the current study, we independently demonstrated the stroke risk of five grades of LASEC, and found a strong positive correlation between the severity of LASEC and stroke incidence.

Fatkin *et al.* had correlated five-grades of LASEC with quantitative VI measurements, and based on their results, we proposed that quantification of LASEC severity could predict stroke outcome in NVAF patients. However, considering that the classification of LASEC into discrete grades of increasing severity may be somewhat arbitrary because of the continuous nature of LASEC (reflected by echogenicity in the LA), and that the semi-quantitative assessment was largely influenced by the experience of the operator^14^, we quantified the severity of LASEC with VI value using color-coded technology. This method allowed us to prospectively determine the VI value of LASEC, as a quantitative stratification method, on the subsequent occurrence of stroke events in patients with NVAF. Compared with the five-grades of LASEC, we found that VI value is a better quantitative method to evaluate the severity of LASEC. More importantly, color-coded images and VI data were all acquired from the software, thus it should be more objective.

The effect of CHADS_2_ on predicting stroke was not significant when the LASEC variable was excluded in our present study, whereas when all variables were included, CHADS_2_ was noted as an independent predictor of stroke. This might be due to the relative small number of patients, particularly on the shortage of the patient with high CHADS_2_ score of 4. Nevertheless, it is well established that the CHADS_2_ score is a simple clinical prediction rule for estimating the risk of stroke in patients with NVAF. A high CHADS_2_ score corresponds to a greater risk of stroke, while a low CHADS_2_ score corresponds to a lower risk of stroke. The CHADS_2_ score has been validated by many studies. However, the CHADS_2_ score may not always be a good predictor of stroke risk, especially in intermediate risk patients and patients with relatively low scores^5^. In our current study, there was no significance achieved in predicting stroke by a CHADS_2_ score ≤2 even all variables included. Earlier reports indicate that in patients with non-valvular AF and a low CHADS_2_ score of 0 or 1, the prevalence of LAT was 3%^20^. Yet, in another study, 10% of persistent AF patients had LAT confirmed by TEE, and 77% of the patients with LAT had a CHADS_2_ score ≤2^18^. Thus, further risk stratification is warranted in these “low to intermediate risk” patients according to the CHADS_2_ score. Our results reveal that the stroke prediction ability of VI value was better than the CHADS_2_ score in patients with NVAF, especially with low to intermediate risk CHADS_2_ score patients. Our study also reveals the similar results with the CHA_2_DS_2_-Vasc score.

Estimating the risk of stroke for individual NVAF patients is crucial in the decision making to provide anticoagulation therapy. While identification of stroke risk in AF is a continuum, prior qualitative or semi-quantitative stroke risk stratification schemes have been used to “artificially” categorize patients into low, moderate, and high risk stroke strata^21^. thereby allowing inherent mistakes in the definition of the real low or high risk patients. On the contrary, the intensity of LASEC reflected by VI value is a continuous variable that may fulfill the demand of continuous assessment of stroke risk^22^. In our study, the quantification of LASEC provided important incremental value in predicting stroke events compared to the CHADS_2_. In addition, our quantitative method is objective and was not affected by the experience of the operators. Despite the potential for LASEC to identify stroke events, this aspect has not been taken into account in stroke risk stratification. Because CHADS_2_ score is easy, safe and can be widely adopted, we propose that CHADS_2_ should be used for all patients with NVAF to define the “high risk” group. Subsequently, for the “low to intermediate risk” patients, we suggest the use of TEE to screen for LASEC, which will further confirm whether an anticoagulant therapy is warranted or not.

### Study limitations

This study was performed in a single dedicated AF ablation center. Therefore, a selection bias could be attributed to the findings, and is a recognized limitation of our study. In addition, the number of patients was relatively small and the follow-up period was relatively short. Thus, a larger multi-center trial with longer follow-up data on stroke event rates is required to validate the usefulness of VI value of LASEC in categorizing the stroke risk in patients with NVAF. Patients with factors that would affect the hemostasis were not included in the study. It is known that hemostasis associated factors, such as fibrinogen level, hematocrit and blood flow, could affect LASEC to a large extent, so the validity of our method in those patient populations is unknown. Furthermore, we did not compare VI value with other quantitative measures due to the lack of an adequate gold standard to assess LASEC severity. Finally, we could not distinguish between paroxysmal and persistent/permanent NVAF, but previous studies suggest that the risk of stroke is similar in these subgroups^23^.

## Conclusions

Quantification of the severity of LASEC with VI value using color-coded technology can improve the predictive ability of LASEC for subsequent stroke events in patients with NVAF. Quantification of LASEC by VI value emerged as the most favorable predictor of stroke; it further identifies the patients who are more likely to undergo aggressive anticoagulant therapy following a high VI value LASEC study. Validation of current findings in a prospective multicenter study with a larger number of patients is required.

## Methods

This study was performed and reported according to the 2010 Statement of Consolidated Standards of Reporting Trials (CONSORT)^24^.

### Ethical Issues

The study protocol was approved by the ethics committee of Nanfang Hospital in Guangzhou. All study participants provided written informed consent. The methods were carried out in accordance with relevant guidelines and regulations.

### Study design and population

This prospective cohort study was conducted during September 2008 to September 2014 at the Nanfang Hospital (Guangzhou, China), a more than 2000 bedded, accredited tertiary care center providing state of the art health care facilities. A total of 780 patients with a diagnosis of NVAF receiving TEE were recruited by searching automated inpatient during September 2008 to September 2012. AF was confirmed by a 12-lead surface electrocardiogram and Holter within a week prior to and during the TEE examination. To reduce the potential selection bias, the exclusion criteria were strictly control. Inclusion criteria were consecutive patients with NVAF undergoing clinically indicated TEE. Exclusion criteria included: (1) a history of rheumatic and congenital heart disease, (2) deep vein thrombosis, (3) patients with mechanical valve, (4) patients with carotid artery plaque, (5) recent (less than four weeks) oral anticoagulant or heparin usage, or (6) patients with conditions such as recent surgery, infection or malignancy, which were likely to affect hemostasis. In addition, patients who underwent radiofrequency ablation or valve replacement surgery after the enrollment were excluded. The cohort was followed up through September 2014, a median follow-up of 2 years (interquartile range 1.4–2.0 years). The follow-up would be ended, if a patient developed stroke. Follow-up information was primarily obtained by telephone interviews.

### Outcomes at follow-up

The primary endpoint in this study was defined as incident stroke events during follow-up. The diagnoses were recorded using the National Stroke Association scoring criteria (1995): focal neurological symptoms persist for 24 h or more, or presence of ischemic or hemorrhagic lesions confirmed using CT or MRI.

### Clinical risk factors of stroke and CHADS_2_ and CHA_2_DS_2_-Vasc score

During hospitalization we obtained clinical parameters comprised age, gender, smoking, history of hypertension, heart failure, diabetes, and anticoagulant or antiplatelet therapy. Laboratory test results included fibrinogen, C-reactive protein, brain natriuretic peptide, uric acid, low-density lipoprotein, etc. Variables required to calculate the CHADS_2_ score included age ≥75 years (1), congestive heart failure (1), hypertension (1), diabetes mellitus (1), and stroke/transient ischemic attack (2)^25^, and the CHA_2_DS_2_-Vasc score included Congestive heart failure (1), Hypertension (1), Age ≥75 (2), Diabetes (1), prior Stroke (2), Vascular disease (1), Age 65–74 (1), Sex category (1)^25^.

### Echocardiography

All patients subsequently underwent transthoracic echocardiography (TTE) and TEE with a Siemens Acuson ultrasound system (Siemens Medical Solutions USA, Inc.). For TTE, a 1.7/3.4-MHz harmonic transducer was used. TEE was performed with a 6.7-MHz multiplane V5Ms transducer, as previously reported^17^. Although higher frequency is associated with more “LASEC”, all images from patients undergoing TEE were recorded at a unified frequency of 6.7 MHz and other ultrasonic parameters were constant through all studies. Measurements were performed off line with syngo^®^ software provided by the manufacturer.

A standardized TTE examination, including the standard imaging planes was performed according to the recommendations of the American Society of Echocardiography^26^. Left ventricular ejection fraction was calculated using the Simpson method, and the left atrial diameter was measured in the M mode. Mitral valve E wave peak velocity was also measured and analyzed.

TEE was performed in the standard manner^27^,^28^,^29^. Written consent was obtained from all patients. Fasting for at least four hours prior to the examination was required for all patients undergoing TEE. Topical lidocaine spray was used for local anesthesia of the oropharynx and if needed, a light sedation with 2.5–5.0 mg midazolam was given intravenously. The LA and its appendage were closely inspected for the presence of LASEC and thrombi by adjustment of the gain settings to an optimised state, which just to be able to distinguish LASEC from electronic background noise. In order to standardise the detection of LASEC in different degrees, overall gain and compress settings were varied throughout their full range. Acoustic frequency and other settings were constant during each study^16^,^18^. Echocardiographic measurements were performed by two independent observers assessing the off-line digitized images. The images were displayed in random order without clinical information of the patient. Inter-observer differences were reconciled by a third observer.

The degree of LASEC was categorized as being either absent (0), mild (1+), mild to moderate (2+), moderate (3+), or severe (4+), on the basis of a grading system described by Fatkin *et al.*^16^ Additionally, color-coded images were acquired by offline image analysis software (MCE version 2.7, University of Virginia, USA) according to the LASEC background subtracted signal placed over the aorticroot, following VI quantification of SEC signal within the region of interest.

### Statistical analysis

The sample size was calculated by taking into account the objectives of the study. Continuous variables were analyzed using independent-Sample *t* tests or one-way ANOVA. Chi-square or Fisher's exact tests were used for categorical variables. Bivariate correlation or logistic regression was performed, and ROC curves generated to compare the predictive power of different stroke stratification methods in the study. All tests were two tailed, and a p-value  < 0.05 was considered statistically significant. Analyses were performed using SPSS software program version 13.0 (SPSS Inc., Chicago, IL, USA).

## Additional Information

**How to cite this article**: Zhao, Y. *et al.* Intensity of Left Atrial Spontaneous Echo Contrast as a Correlate for Stroke Risk Stratification in Patients with Nonvalvular Atrial Fibrillation. *Sci. Rep.* **6**, 27650; doi: 10.1038/srep27650 (2016).

## Supplementary Material

Supplementary Information

## Figures and Tables

**Table 1:** Baseline clinical and echocardiographic characteristics according to stroke event (primary endpoint).

	Stroke (n = 20)	Non-stroke (n = 186)	p value
Demographics			
Male	11 (55.0%)	129 (69.4%)	0.192
Age, y	54.90 ± 11.52	53.85 ± 11.87	0.258
Clinical data			
Smoking	5 (25.0%)	35 (18.8%)	0.552
DM	4 (20.0%)	27 (14.5%)	0.529
Hypertension	13 (65.0%)	132 (71.0%)	0.579
Hyperlipidemia	6 (30.0%)	40 (21.5%)	0.401
CAD	1 (5.0%)	16 (8.6%)	0.553
HF	6 (30.0%)	41 (22.0%)	0.434
NPAF	11 (55.0%)	65 (34.9%)	0.077
EF	63.31 ± 9.18	63.46 ± 8.19	0.937
LAD	42.85 ± 6.10	42.73 ± 7.62	0.946
LASEC	15 (75.0%)	68 (36.6%)	0.001*
LAT	7 (35.0%)	10 (5.4%)	0.000*
CHADS_2_	1.50 ± 1.10	0.87 ± 0.85	0.022*
CHA_2_DS_2_-Vasc	2.65 ± 1.42	1.58 ± 1.25	0.000*
Anticoagulant drugs use			
Warfarin	11 (55.0%)	82 (44.1%)	0.351

DM: Diabetes mellitus; CAD: Coronary artery disease; HF: Heart failure; NPAF: Non-paroxysmal atrial fibrillation (including persistent atrial fibrillation and permanent atrial fibrillation); EF: Ejection fraction; LAD: Left atrial diameter; LASEC: Left atrial spontaneous echo contrast; LAT: Left atrial thrombus; CHADS_2_: Congestive heart failure (1), Hypertension (1), Age ≥75 (1), Diabetes (1), prior Stroke (2); CHA_2_DS_2_-Vasc: Congestive heart failure (1), Hypertension (1), Age ≥75 (2), Diabetes (1), prior Stroke (2), Vascular disease (1), Age 65–74 (1), Sex category(1). *p < 0.05.

**Table 2:** Results of the multivariate logistic regression models showing the independent predictors for stroke.

Variable	Model 1	Model 2	Model 3	Model 4
	p value, OR (95% CI)	p value, OR (95% CI)	p value, OR (95% CI)	p value, OR (95% CI)
Smoking	0.464, 0.60 (0.15–2.36)	0.254, 0.42 (0.10–1.86)	0.303, 0.44 (0.09–2.09)	0.401, 0.53 (0.12–2.32)
AF	0.169, 2.18 (0.72–6.60)	0.490, 1.52 (0.46–5.03)	0.435, 0.52 (0.10–2.68)	0.666, 0.73 (0.17–3.11)
CAD	0.730, 0.65 (0.06–7.51)	0.666, 0.58 (0.05–6.81)	0.550, 0.45 (0.03–6.30)	0.559, 0.48 (0.04–5.77)
CRP	0.744, 0.99 (0.96–1.03)	0.727, 1.01 (0.97–1.04)	0.832, 1.01 (0.96–1.05)	0.998, 1.00 (0.96–1.04)
Fibrinogen	0.775, 1.10 (0.57–2.14)	0.986,1.01 (0.53–1.93)	0.955, 0.98 (0.474–2.02)	0.795, 0.91(.46–1.81)
BNP	0.241, 1.00 (1.00–1.001)	0.419, 1.00 (1.00–1.001)	0.982,1.00 (1.00–1.001)	0.793, 1.00 (1.00–1.001)
BUA	0.747, 1.00 (1.00–1.01)	0.948, 1.00 (0.99–1.01)	0.990, 1.00 (0.99–1.01)	0.784, 1.00 (1.00–1.01)
LDL	0.960, 1.02 (0.54–1.93)	0.716, 1.02 (0.44–1.75)	0.498, 0.76 (0.35–1.67)	0.641, 0.84 (0.39–1.79)
EF	0.705, 0.99 (0.92–1.06)	0.935, 1.00 (0.93–1.07)	0.579, 1.02 (0.95–1.11)	0.855, 1.01 (0.94–1.09)
LA	0.329, 0.96 (0.89–1.04)	0.083, 0.93 (0.85–1.01)	0.046, 0.91 (0.83–1.00)	0.096, 0.93 (0.85–1.01)
MVe	0.676, 1.00 (0.97–1.02)	0.421, 0.99 (0.96–1.02)	0.454, 0.99 (0.96–1.02)	0.686, 0.99 (0.97–1.02)
LAT	**0.007**, 6.28 (1.66–23.79)	0.069, 3.68 (0.90–14.98)	0.586, 1.57 (0.31–7.98)	0.609, 1.53 (0.30–7.85)
CHADS_2_	0.090	**0.047**	**0.025**	**0.037**
CHADS_2_(score 1)	0.519, 0.62 (0.15–2.65)	0.542, 0.63 (0.14–2.81)	0.332, 0.44 (0.08–2.33)	0.401, 0.50 (0.10–2.51)
CHADS_2_(score 2)	0.076, 3.72 (0.87–15.93)	0.047, 4.75 (1.02–22.06)	0.055, 5.27 (0.96–28.82)	0.071, 4.12 (0.88–19.25)
CHADS_2_(score 3)	0.096, 4.39 (0.77–25.11)	**0.044**, 6.86 (1.06–44.53)	**0.014**, 13.891 (1.70–113.33)	**0.026**, 8.49 (1.29–55.92)
CHADS_2_(score 4)	1.000, 0.00	1.000, 0.00	1.000, 0.00	1.000, 0.00
VI value				**0.002**, 1.09 (1.03–1.15)
Qualitative LASEC		**0.014**, 6.59 (1.46–29.71)		
Graded LASEC			**0.034**	
LASEC grade 1			0.112, 5.16 (0.68–39.16)	
LASEC grade 2			0.071, 7.23 (0.85–61.73)	
LASEC grade 3			**0.004**, 45.32 (3.37–608.69)	
LASEC grade 4			**0.002**, 157.98 (6.05–4127.14)	
Constant	0.585, 0.16	0.987,0.95	0.808, 2.78	0.957, 1.24

AF: atrial fibrillation; CAD: coronary artery disease; CRP: C reactive protein; BUA: blood uric acid; EF: ejection fraction; LA: left atrium; MVe: Mitral valve E; LAT: left atrial thrombus. CHADS_2_: Congestive heart failure (1), Hypertension (1), Age ≥75 (1), Diabetes (1), prior Stroke (2). For model 1, Smoking, AF, CAD, CRP, fibrinogen, BNP, BUA, LDL, EF, LA, MVe, LAT, and CHADS_2_ were all entered into this multivariable model. For model 2, model 1 plus qualitative LASEC were all entered into this multivariable model. For model 3, model 1 plus graded LASEC were all entered into this multivariable model. For model 4, model 1 plus VI value of LASEC were all entered into this multivariable model.

**Table 3:** Area under the ROC curve (AUC) of five stroke predictor variables.

Predictor variables	AUC (mean ± SE)	p	95% CI
VI value	0.844 ± 0.041	0.000	0.764–0.924
Graded LASEC	0.754 ± 0.065*	0.000	0.627–0.881
CHA_2_DS_2_–Vasc	0.720 ± 0.065#	0.001	0.592–0.848
Qualitative LASEC	0.692 ± 0.060*	0.005	0.574–0.810
CHADS_2_	0.668 ± 0.073^#^	0.014	0.525–0.811
Left Atrial Thrombus	0.648 ± 0.074*	0.030	0.502–0.794

*p < 0.01 *vs.* VI value, ^#^p < 0.05 *vs.* VI value.
