# Supplementary material for: Intensity of Left Atrial Spontaneous Echo Contrast as a Correlate for Stroke Risk Stratification in Patients with Nonvalvular Atrial Fibrillation
Source: Sci Rep. 2016 Jun 9;6:27650. doi: 10.1038/srep27650 (PMC4899705; doi:10.1038/srep27650)
Supplement: Supplementary Information [file srep27650-s1.pdf]

Intensity of Left Atrial Spontaneous Echo Contrast as a Correlate for Stroke Risk Stratification in Patients with Nonvalvular Atrial Fibrillation

Yuanping Zhao[#1,2]; Lijing Ji[#3]; Jian Liu[#1]; Juefei Wu[#1]; Yan Wang[1]; Shuxin Shen[1]; Shengcun Guo[1]; Rong Jian[1]; Gangbin Chen[1]; Xuan Wei[1]; Wangjun Liao[4]; Shelby Kutty[5]; Yulin Liao[1]; Jianping Bin*[1]

[1]State Key Laboratory of Organ Failure Research, Department of Cardiology, Nanfang Hospital, Southern Medical University, Guangzhou, China; [2]Department of Gerontology, Cangzhou Central Hospital, Cangzhou, china. [3]Department of Ultrasonography, and [4]Department of Oncology, Nanfang Hospital, Southern Medical University, Guangzhou, China. [5]University of Nebraska Medical Center, Children's Hospital and Medical Center, Omaha, NE, United States.

[#]These authors contributed equally to this work.

Table S1. Results of the multivariate logistic regression models showing the independent predictors for stroke.

Variable	Model 1	Model 2	Model 3	Model 4
	p value, OR (95%CI)	p value, OR (95%CI)	p value, OR (95%CI)	p value, OR (95%CI)
Smoking	0.814, 1.18(0.30-4.69)	0.884, 0.89(0.20-3.99)	0.966, 0.97(0.22-4.31)	0.941, 1.06(0.24-4.56)
AF	0.362, 1.71(0.54-5.42)	0.913, 1.07(0.30-3.86)	0.421, 0.51(0.10-2.65)	0.526, 0.61(0.13-2.85)
CAD	0.853, 0.80(0.08-8.30)	0.845, 0.79(0.08-8.01)	0.702, 0.62(0.05-7.26)	0.716, 0.64(0.06-6.87)
CRP	0.976, 1.00(0.97-1.04)	0.661, 1.01(0.97-1.04)	0.822, 1.00(0.97-1.04)	0.957, 1.00(0.96-1.04)
Fibrinogen	0.927, 1.03(0.50-2.14)	0.834, 0.92(0.45-1.92)	0.819, 0.91(0.42-2.00)	0.655, 0.84(0.39-1.81)
BNP	0.126, 1.00(1.00-1.001)	0.206, 1.00(1.00-1.001)	0.336, 1.00(1.00-1.001)	0.262, 1.00(1.00-1.001)
BUA	0.894, 1.00(0.99-1.01)	0.608, 1.00(0.99-1.00)	0.468, 1.00(0.99-1.00)	0.725, 1.00(0.99-1.00)
LDL	0.659, 0.85(0.42-1.74)	0.563, 0.80(0.38-1.70)	0.674, 0.85(0.39-1.82)	0.709, 0.86(0.40-1.86)
EF	0.783, 0.99(0.92-1.06)	0.993, 1.00(0.93-1.08)	0.807, 1.01(0.93-1.09)	0.926, 1.00(0.93-1.08)
LA	0.759, 0.99(0.91-1.07)	0.309, 0.96(0.88-1.04)	0.279, 0.95(0.87-1.04)	0.361, 0.96(0.88-1.05)
MVe	0.184, 0.98(0.95-1.01)	0.148, 0.98(0.95-1.01)	0.212, 0.98(0.95-1.01)	0.345, 0.99(0.95-1.02)
LAT	**0.015**, 5.7(1.40-23.17)	0.056, 4.10(0.97-17.44)	0.177, 2.96(0.61-14.33)	0.363, 2.18(0.41-11.74)
CHA_2DS_2-Vasc	0.042	0.050	0.075	0.094
CHA_2DS_2-Vasc (score 1)	0.735, 0.69(0.08-5.80)	0.764, 0.72(0.08-6.24)	0.560, 0.52(0.06-4.71)	0.572, 0.55(0.07-4.45)
CHA_2DS_2-Vasc (score 2)	0.427, 2.17(0.32-14.60)	0.547, 1.81(0.26-12.60)	0.771, 1.34(0.19-9.55)	0.650, 1,54(0.24-9,79)
CHA_2DS_2-Vasc (score 3)	0.689, 1.51(0.20-11.2)	0.789, 1.32(0.17-10.24)	0.999, 1.00(0.11-9.35)	0.922, 0.90(0.10-7.81)
CHA_2DS_2-Vasc (score 4)	**0.004**, 26.67(2.79-254.92)	**0.004**, 26.79(2.81-255.33)	**0.010**, 20.61(2.09-203.09)	**0.010**, 17.76(1.97-159.92)
CHA_2DS_2-Vasc (score 5)	0.221, 6.73(0.32-142.21)	0.211, 7.42(0.32-171.55)	0.510, 3.06(0.11-85.47)	0.638, 2.20(0.08-58.86)
CHA_2DS_2-Vasc (score 6)	0.999, 0.00	0.999, 0	0.999, 0	0.999, 0
VI value				**0.009**,1.07(1.02-1.13)
Qualitative LASEC		**0.036**, 5.51(1.12-27.10)		
Graded LASEC			0.111	
LASEC grade 1			0.188, 3.68(0.53-25.64)	
LASEC grade 2			0.111, 5.51(0.67-45.04)	
LASEC grade 3			**0.008**, 24.33(2.32-255.22)	
LASEC grade 4			**0.028**, 24.48(1.41-425.35)	
Constant	0.754, 0.34	0.864, 1.92	0.743, 3.81	0.881, 1.80

AF: atrial fibrillation; CAD: coronary artery disease; CRP: C reactive protein; BUA: blood uric acid; EF: ejection fraction; LA: left atrium; MVe: Mitral valve E; LAT: left atrial thrombus. CHA_2DS_2-Vasc: Congestive heart failure (1), Hypertension (1), Age ≥75 (2), Diabetes (1), prior Stroke (2), Vascular disease (1), Age 65-74 years (1), Sex category (1).

For model 1, Smoking, AF, CAD, CRP, fibrinogen, BNP, BUA, LDL, EF, LA, MVe, LAT, and CHA_2DS_2VASc were all entered into this multivariable model.

For model 2, model 1 plus qualitative LASEC were all entered into this multivariable model.

For model 3, model 1 plus graded LASEC were all entered into this multivariable model.

For model 4, model 1 plus VI value of LASEC were all entered into this multivariable model.
